# Intraductal papillary mucinous neoplasm with gastric penetration diagnosed using endoscopic imaging and aspiration cytology

**DOI:** 10.1055/a-2615-6391

**Published:** 2025-06-26

**Authors:** Koichi Soga, Fuki Hayakawa, Mayumi Yamaguchi, Yo Fujimoto, Ryosaku Shirahashi, Ikuhiro Kobori, Masaya Tamano

**Affiliations:** 1 26263 Department of Gastroenterology, Dokkyo Medical University Saitama Medical Center, Koshigaya, Saitama, Japan

Intraductal papillary mucinous neoplasms (IPMNs) are cystic epithelial tumors arising in the pancreatic ductal system and characterized by intraductal papillary proliferation of mucin-producing neoplastic cells 1 . One rare complication of IPMNs is the formation of fistulas in adjacent organs, including the gastrointestinal tract. Cytological examination plays a pivotal role in the differential diagnosis of pancreatic cystic lesions, guiding clinical decision making and therapeutic strategies 2 .

A 48-year-old man with a history of chronic pancreatitis underwent pancreaticoduodenectomy 4 years before presentation. Follow-up abdominal imaging confirmed dilation of the remnant pancreatic duct suspected to be associated with malignant IPMN (mIPMN) and intragastric penetration of the IPMN through the pancreatic duct ( Fig. 1 ). We decided to perform an endoscopic procedure to examine the worsening IPMN.

**Fig. 1:**
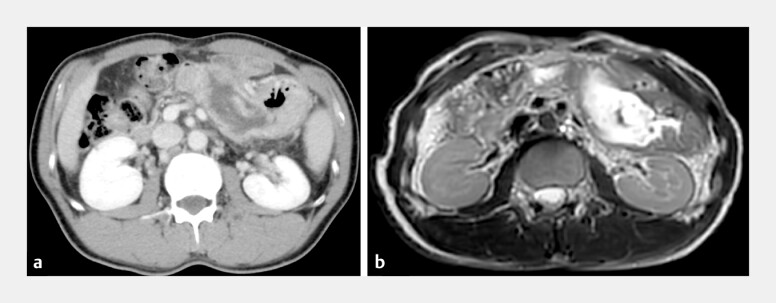
Computed tomography (CT) and magnetic resonance imaging (MRI) of gastric penetration by intraductal papillary mucinous neoplasms (IPMNs). Contrast-enhanced CT (CE-CT) revealed a markedly dilated main pancreatic duct (MPD) and thin pancreatic tissue. **a** The tumor in the tail extended outside the pancreas, suggesting invasion of the stomach. **b** MRI confirmed the dilation of the MPD initially observed on CE-CT, with intraductal tumors with low signal intensity on T2-weighted images (T2-WI). The wall of the MPD and fistula showed high signal intensity on T2-WI.

Esophagogastroduodenoscopy revealed abundant mucus discharge from the stomach penetration site. We performed cytological analysis using endoscopic aspiration to collect the secreted mucus. Aspiration was performed using a material collection tube connected to an endoscope ( Fig. 2 ). After sufficient aspiration of a large amount of mucus, the papillary structure of the mIPMN was observed endoscopically.

**Fig. 2:**
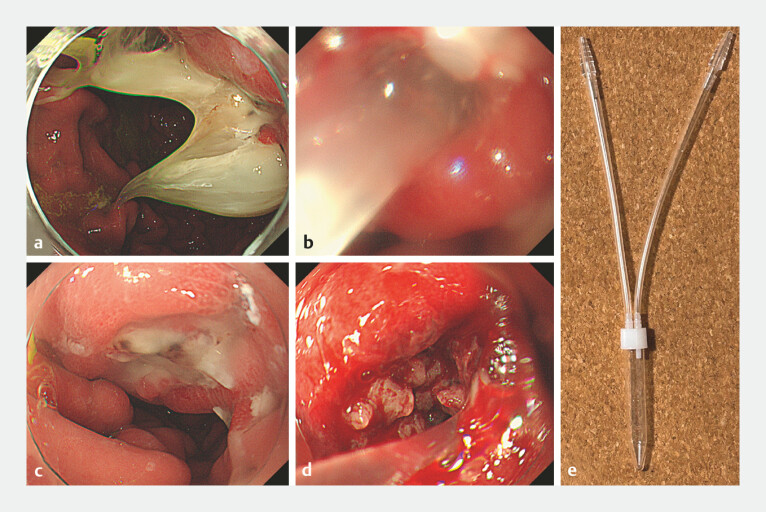
Endoscopic imaging of intraductal papillary mucinous neoplasms (IPMNs) with gastric penetration and mucus discharge. **a** Esophagogastroduodenoscopy revealed gastropancreatic fistulas on the posterior wall of the gastric upper body and mucus discharged from the fistulas. **b** For cytological analysis, mucus was collected by endoscopic aspiration. **c** After sufficient aspiration and mucus removal using an endoscope, the stomach and pancreatic fistulas were observed endoscopically. **d** Insertion of the endoscope into the fistula revealed a papillary raised tumor. **e** The collection tube was connected to the endoscope under suction (SB-KAWASUMI, Kanagawa, Japan).

Approximately 30 mL of aspirated sample was subjected to cytological examination, revealing atypical glandular cells and abundant mucus adhesions (Papanicolaou stain, ×200). Biopsy of the papillary tumor performed after adequate mucus aspiration also showed cells with enlarged chromatin-dense nuclei. These findings were consistent with adenocarcinoma (hematoxylin and eosin stain, ×200). Based on these findings, mIPMN of the pancreas with a fistula penetrating the stomach was confirmed ( Fig. 3 , Video 1 ).

**Fig. 3:**
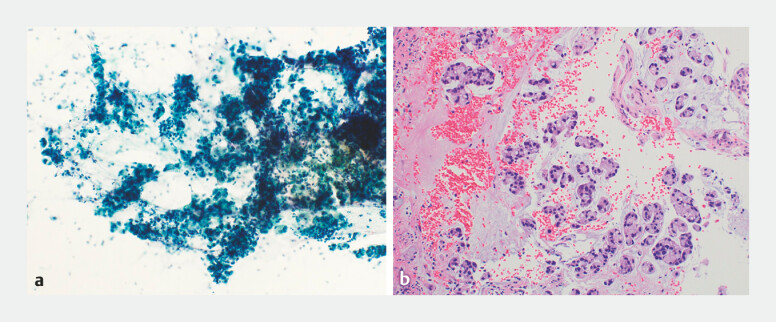
Cytological findings from aspirated mucus collected using endoscopy. **a** Collection of aspirated mucus revealed atypical mucus-covered glandular cells (Papanicolaou stain, ×200). **b** Biopsies of the papillary tumor performed after adequate mucus aspiration showed large and small clusters of tumor cells with enlarged chromatin-rich nuclei and pale acidophilic or foamy cytoplasm, accompanied by extracellular mucus; these findings were consistent with adenocarcinoma (hematoxylin and eosin stain, ×200).

Intraductal papillary mucinous neoplasm with gastric penetration diagnosed using endoscopic imaging and aspiration cytology.Video 1

In the present case, mIPMN penetrating the stomach was successfully diagnosed using aspiration cytology and endoscopy. Endoscopic imaging clearly depicted penetration of the IPMN into the gastric cavity, associated with significant mucus discharge. This case provides valuable insights into the diagnostic approach for this rare complication of IPMN and enhances our understanding of these neoplasms.

Endoscopy_UCTN_Code_CCL_1AZ_2AB

## Notes

Endoscopy E-Videos https://eref.thieme.de/e-videos
*E-Videos* is an open access online section of the journal *Endoscopy* , reporting on interesting cases and new techniques in gastroenterological endoscopy. All papers include a high-quality video and are published with a Creative Commons CC-BY license. Endoscopy E-Videos qualify for HINARI discounts and waivers and eligibility is automatically checked during the submission process. We grant 100% waivers to articles whose corresponding authors are based in Group A countries and 50% waivers to those who are based in Group B countries as classified by Research4Life (see: https://www.research4life.org/access/eligibility/ ). This section has its own submission website at https://mc.manuscriptcentral.com/e-videos .
